# Multimodality Radiologic and Pathologic Findings in Large B-cell Lymphoma With Hepatic Presentation in an Unusually Young Male

**DOI:** 10.7759/cureus.66847

**Published:** 2024-08-14

**Authors:** Konrad Drzymalski, Alexandria Sobczak, Brianna Castellano, Lexie Leon, Alejandro Biglione

**Affiliations:** 1 Medicine, Dr. Kiran C. Patel College of Osteopathic Medicine, Nova Southeastern University, Fort Lauderdale, USA; 2 Internal Medicine, Wellington Regional Medical Center, Wellington, USA

**Keywords:** extranodal diffuse large b-cell lymphoma, clinical case report, non-hodgkins lymphoma, hepatic lymphoma, large b cell lymphoma

## Abstract

Diffuse large B-cell lymphoma (DLBCL) is the most prevalent type of non-Hodgkin lymphoma (NHL), and typically presents in patients who are at least 60 years old with gastrointestinal (GI) tract involvement. We report a case of a young patient with DLBCL. A 27-year-old African American male presented to the emergency room with complaints of abdominal distention. Imaging showed hepatosplenomegaly with multiple nodular lesions in both the liver and spleen. The biopsy confirmed a diagnosis of DLBCL. This case report highlights a rare clinical presentation of DLBCL due to the uncommon hepatic initial presentation of the disease paired with the patient’s age and race varying significantly from the demographic norm. Clinicians should maintain a high index of suspicion for DLBCL in patients with atypical extranodal involvement, such as in this patient, to optimize patient outcomes.

## Introduction

Diffuse large B-cell lymphoma (DLBCL) is the most prevalent type of non-Hodgkin lymphoma (NHL), representing approximately 25-30% of all cases [1]. The clinical presentation typically involves a rapidly enlarging mass in the neck or abdomen. In addition, about 30% of patients with DLBCL exhibit systemic "B" symptoms, including fever, weight loss, and night sweats [2]. The gastrointestinal (GI) tract is the most common site of extranodal involvement in DLBCL, making the secondary involvement of the liver in this young patient unique [2]. DLBCL is an aggressive disease. However, it can be cured in more than 60% of the patients, and hence it is important to have a high index of suspicion in patients with atypical extranodal involvement such as the patient described in this case report [3]. In addition, the median age at presentation of DLBCL is 64 years, with the highest incidence in non-Hispanic White men, making this case of a 27-year-old African American male an uncommon presentation [1]. We discuss the clinical presentation, diagnosis, and management of DLBCL.

## Case presentation

A 27-year-old African American male presented to the emergency room with complaints of abdominal distention and diffuse aching abdominal pain for the past few weeks. His review of symptoms was positive for night sweats, nightly fevers up to 38 ℃, and weight loss of 11 pounds in the past three months. He had a significant social history of alcohol use, commonly drinking 0.5-1 liters of liquor per day on the weekends. He denied tobacco or marijuana use. He was sexually active with one partner with whom he practiced safe sex. His family history was unremarkable.

On admission, the patient's vital signs included a temperature of 37.1 ℃, respiratory rate of 18 breaths per minute, oxygen saturation of 94% on room air, and a blood pressure of 117/73 mmHg. On physical examination, his lungs were clear to auscultation and his heart was at regular rate and rhythm. No jaundice or pallor was present. He had a palpable axillary lymph node on the right side. The abdomen was distended, soft, and non-tender with positive bowel sounds on auscultation, and was tympanic on percussion. A complete blood count and metabolic panel were taken on admission (Tables 1-2). Table 3 presents the initial liver function tests. The HIV and hepatitis panels were both negative (Table 4). Initial labs were evaluated based on the institution’s established reference ranges.

**Table 1:** Initial complete blood count (CBC)

Lab	Initial value	Normal range
White blood cell count (cells/mcL)	2,400	4,500 - 10,500
Red blood cell count (cells/mcL)	3,160,000	4,400,000 - 6,150,000
Hemoglobin (gm/dL)	9.0	14.0 - 18.0
Hematocrit (%)	25.7	40.0 - 54.0
Mean corpuscular volume (fL)	81.3	81.0 - 96.0
Mean corpuscular hemoglobin (pg)	28.5	27.0 - 34.0
Platelet count (platelets/mcL)	101,000	150,000 - 450,000

**Table 2:** Initial complete metabolic panel (CMP)

Lab	Initial value	Normal range
Glucose (mg/dL)	144	74 - 106
Sodium (mmol/L)	132	135 - 148
Potassium (mmol/L)	3.7	3.6 - 5.2
Chloride (mmol/L)	102	95 - 110
Bicarbonate (mEq/L)	26	21 - 32
Blood urea nitrogen (mg/dL)	12	7 - 18
Creatinine (mg/dL)	0.76	0.7 - 1.3

**Table 3:** Initial liver function tests

Lab	Initial value	Normal range
Calcium (mg/dL)	7.4	8.5 - 10.2
Albumin (g/dL)	2.0	3.4 - 5.0
Total protein (g/dL)	4.5	6.4 - 8.2
Total bilirubin (mg/dL)	1.2	0 - 1
Alkaline phosphatase (U/L)	1,385	45 - 117
Aspartate aminotransferase (U/L)	375	15 - 37
Alanine aminotransferase (U/L)	209	12 - 78

**Table 4:** HIV and hepatitis panel

Lab	Patient value	Reference value
Hepatitis B surface antigen	Negative	Negative
Hepatitis B surface antibody	Positive	Negative
Hepatitis B core antibody	Negative	Negative
Human immunodeficiency virus I and II antibodies	Negative	Negative

**Figure 1:**
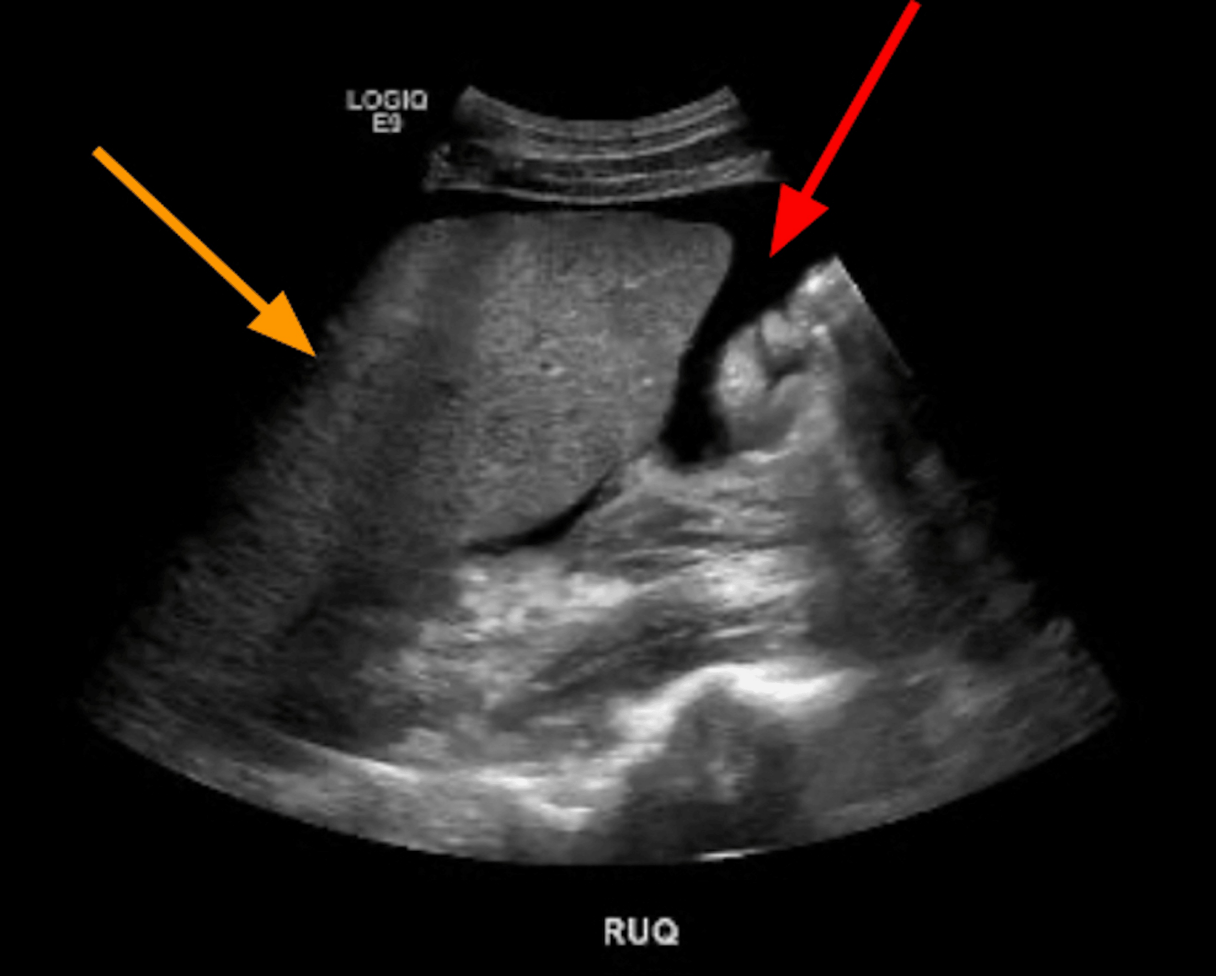
Complete abdominal ultrasound The image displays excessive abdominal ascites (red arrow), with hepatomegaly also observed (orange arrow)

**Figure 2:**
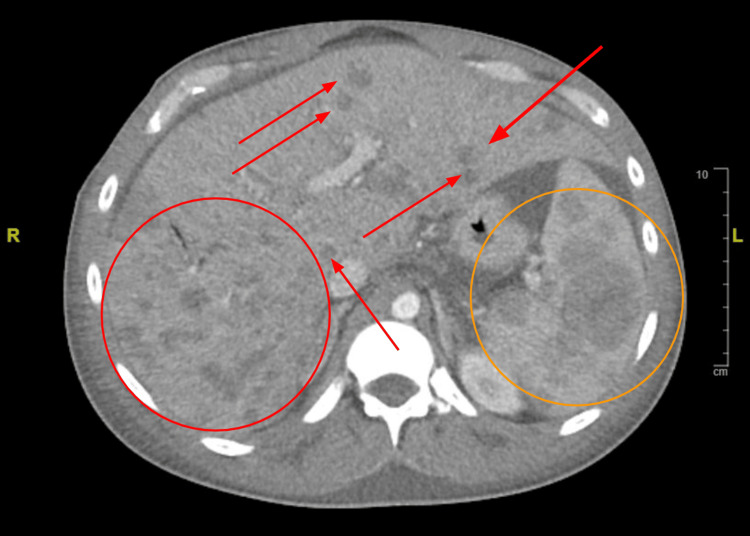
CT chest/abdomen/pelvis with IV contrast (axial view) The image displays multiple hepatic nodules and metastatic disease involving the liver and spleen (spleen seen inside the orange circle, hepatic nodules notated with red circles and arrows) CT: computed tomography

**Figure 3:**
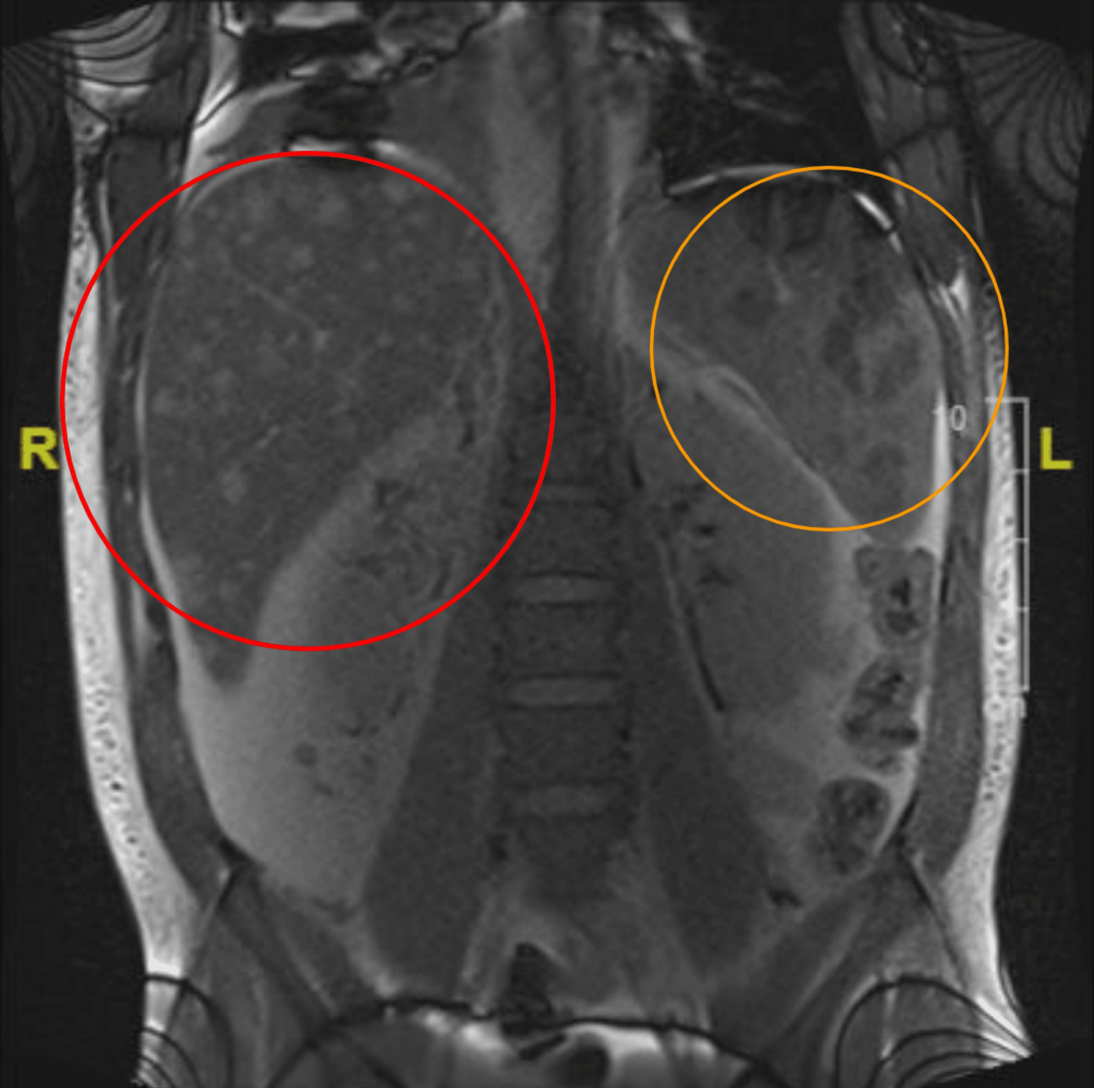
MRI without contrast (coronal view) The image displays hepatosplenomegaly with multiple nodular lesions in both liver (red circle) and spleen (orange circle) MRI: magnetic resonance imaging

Ultrasound of the abdomen revealed diffuse abdominal ascites and hepatosplenomegaly (Figure 1). CT of the abdomen showed numerous hypodense lesions through the liver and spleen measuring up to 4.7 cm in diameter (Figure 2). These findings raised concerns about metastasis to the liver, spleen, axillary lymph nodes, and supraclavicular lymph nodes. Magnetic resonance cholangiopancreatography (MRCP) without contrast was utilized to further explore the source of his abdominal pain with resultant imaging showing hepatosplenomegaly with multiple nodular lesions in both the liver and spleen (Figure 3). Extensive peritoneal ascites and bilateral pleural effusions were also visualized.

**Figure 4:**
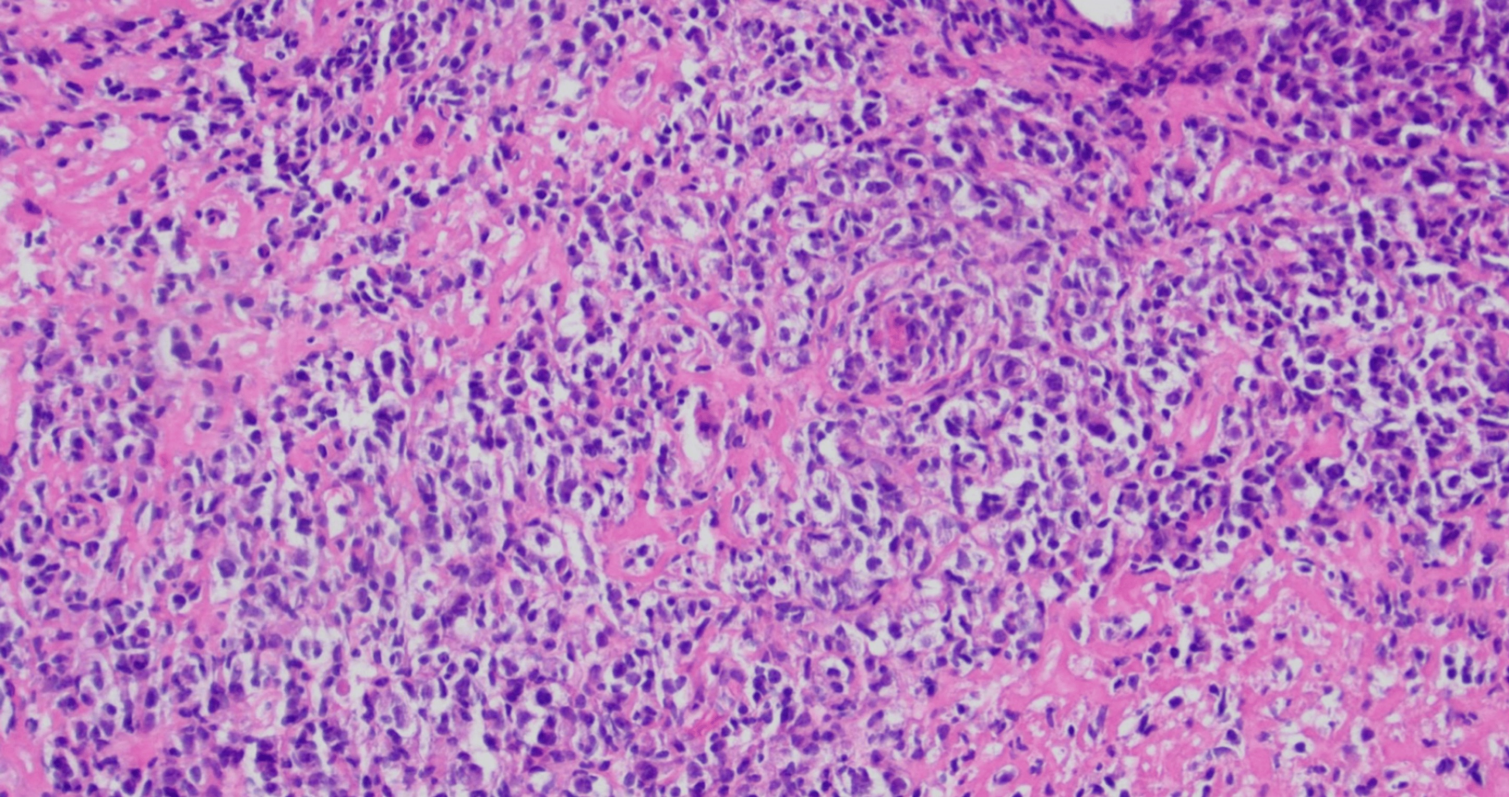
Hematoxylin and eosin stain of right axillary lymph node biopsy The image displays markedly pleomorphic mononuclear cells with focally abundant vacuolated cytoplasm and/or retraction artifact

Paracentesis of peritoneal fluid performed by interventional radiology showed chronic lymphocytic inflammatory effusion and no malignant cells. A fine needle biopsy of the liver showed atypical cells suspicious for large B-cell lymphoma. A core biopsy of the aforementioned palpable axillary lymph node was also completed. Subsequent immunohistochemical studies were performed (Figure 4). The axillary lymph node biopsy showed many large atypical lymphocytes positive for CD20, PAX-5, CD30, CD23, BCL-6, and MUM-1. The atypical cells were negative for CD3, CD5, CD10, BCL-2, CD15, CD4, CD7, and CD8. 

Following diagnosis, the patient was started on cyclophosphamide, doxorubicin, vincristine, and prednisone (CHOP) plus rituximab inpatient. A treatment plan was established for the patient to continue chemotherapy treatment every three weeks for at least six cycles in an outpatient setting.

## Discussion

DLBCL is the most common form of NHL, with approximately 25,000 new cases diagnosed each year [4]. As per the United States cancer registry, the incidence rate of DLBCL is approximately 7.2 per 100,000 [5]. While DLBCL can occur in childhood, it primarily affects non-Hispanic White adults with a peak incidence in individuals over the age of 60 years, with a median age at diagnosis of 64 and a slight predominance in the male sex [1]. The etiology underlying DLBCL is both complex and multifactorial and presents as an amalgamation of several different underlying mechanisms. The most prominent of these mechanisms involve genetic factors, immunodeficiencies, chemical exposures, chronic inflammation, and even infectious agents [6]. The complex heterogeneity of DLBCL can be attributed to the combination of these aforementioned underlying elements.

As indicative of their names, B-cell lymphomas result from the malignant transformation and resultant proliferation of B cells and are largely comprised of germinal center B-cells (GCBs) [1]. These GCBs serve as the cell-of-origin in modern pathology reports and subsequent gene profiling and immunohistochemical analysis can aid with the diagnosis. In terms of immunohistochemical stain patterns and genetic profiling, DLBCL B-cells exist in two main molecular subtypes based on their aforementioned staging and molecular expression: GCB-like and activated B-cell-like (ABC) subtypes [1].

Typical DLBCL cells in the GCB subtype exhibit genetic and immunohistochemical profiles resembling normal germinal center B-cells, with enhanced expression of CD10 and BCL-6 markers with lower expression of MUM1/IRF4 [7]. The GCB subtype is further associated with a more favorable prognosis with better responses to treatment. On the other hand, typical DLBCL cells in the ABC subtype exhibit profiles more suggestive of activated B-cells rather than germinal center B-cells, with increased expression of MUM1/IRF4 markers and decreased CD10 and BCL-6 expression [7]. As compared to GCB, the ABC subtype is associated with poorer prognoses and tends to be resistant to standard treatments sought to address the GCB subtype [8]. 

As the majority of NHLs spread hematogenously, DLBCL most commonly presents in the advanced stages of the disease. While up to 30% of patient presentation involves classic “B” symptoms, which include fatigue, fever, weight loss, and profuse night sweats, the diffuse advancement of the disease process on initial presentation calls for a thorough lymphoid investigation as well as evaluation of hepatic or splenic enlargement which are also common [1]. Approximately 50% of patients have extranodal involvement, with the GI tract serving as the primary extranodal site of spread [1]. Patient presentation is also further dictated by the pathognomonic signs related to the organ that serves as the diseased extranodal site. Therefore, secondary involvement of the liver in patients with DLBCL may present with jaundice, an infiltrative or obstructive pattern of liver enzyme abnormalities, ascites, and radiological findings indicative of liver disease shrouding the initial diagnosis of a malignancy.

Further diagnostic workup of DLBCL can be done via imaging modalities such as ultrasound, CT, or MRI. Signs of lymphadenopathy are a hallmark of identification, involving rounded or oval masses most commonly seen in the neck, axilla, and abdomen. Furthermore, abdominal involvement of this pathology commonly presents as fluid collections with peritoneal thickening or distinct organomegaly of the liver and spleen [9]. Approximately two-thirds of patients initially presenting with DLBCL are classified as having advanced-stage disease, more specifically a corresponding diagnosis of Stage III or IV DLBCL per the Lugano Classification System [10]. Stage I and II, also known as limited stage disease, indicate the involvement of one to two nodes, or groupings of small adjacent nodes, with limited or no extranodal involvement. The aforementioned advanced-stage disease classification for Stages III and IV indicates nodal involvement both above and below the diaphragm while also including the spleen, with additional noncontiguous lymphatic involvement [10].

The management of these advanced-stage patients involves cycles of immunotherapy and chemotherapy individually catered to patients depending on their specific neoplasm type. Rituximab is a common agent utilized in DLBCL therapy and thorough management and prevention of tumor lysis syndrome and or viral reactivation must be considered [10]. Rituximab is most commonly combined with multi-drug therapy regimens involving other agents such as cyclophosphamide, doxorubicin, vincristine, and prednisone, although as previously mentioned, multiple combinations exist and each is carefully selected for the individual treatment [10]. Following treatment cycles, PET and CT imaging studies are conducted to monitor the neoplasm with routine follow-up visits scheduled every three months for the first year to continue monitoring treatment progress. 

This case report thus highlights a rare and atypical presentation of DLBCL in a 27-year-old African American male. Firstly, this report seeks to underscore the diagnostic challenges posed by this disease process, especially in young patients who do not conform to the usual demographic characteristics of this condition. The patient’s history of long-term alcohol abuse along with nonspecific abdominal symptoms on initial presentation led to the difficulty in identifying DLBCL, necessitating a thorough workup for proper diagnosis. Thus, distinguishing between primary hepatic disease and secondary involvement by DLBCL is imperative in guiding appropriate treatment strategies. The thorough investigative process for proper diagnosis is most highlighted through the multiple imaging modalities and biopsy procedures conducted to confirm the diagnosis of DLBCL, where history and physical alone are not sufficient. The identification of large atypical lymphocytes positive for BCL-6, MUM-1, and CD20 with the absence of other aforementioned markers allowed for the confirmation of diagnosis and distinction from other lymphoid malignancies. 

This case exemplifies the aggressive nature of DLBCL and its propensity for metastasis, further highlighting the importance of prompt diagnosis and initiation of appropriate treatment for patients with such a diagnosis. However, despite its aggressiveness, DLBCL is potentially curable in more than 60% of patients who achieve remission with frontline therapy [3,11]. Therefore, maintaining a high index of suspicion for DLBCL in patients with atypical extranodal involvement, such as in this patient, is crucial for optimizing patient outcomes. Early recognition of DLBCL and timely initiation of treatment are essential to improve survival rates and minimize disease-related morbidity and mortality, given the severity of the disease and poor prognosis associated with undetected diffuse malignancy.

## Conclusions

DLBCL is most commonly seen in non-Hispanic White patients over the age of 60 years. It typically presents with an enlarging mass in the neck or abdomen and metastasizes to the GI tract. In this case, DLBCL was found in a young African American male with secondary liver involvement. The patient’s history of long-term alcohol abuse along with nonspecific abdominal symptoms on initial presentation made identifying DLBCL challenging. DLBCL has a high rate of recovery if diagnosed early. Therefore, it is important to always consider DLBCL as a differential in young patients with liver failure and lymph node or B-cell symptoms to maximize their chances of recovery.
